# Oxidation of 1-propanol to propionic acid with hydrogen peroxide catalysed by heteropolyoxometalates

**DOI:** 10.1186/s13065-021-00750-5

**Published:** 2021-04-01

**Authors:** Minxue Liu, Fengli Yu, Bing Yuan, Congxia Xie, Shitao Yu

**Affiliations:** 1 State Key Laboratory Base of Eco-Chemical Engineering, College of Chemistry and Molecular Engineering, Qingdao University of Science and Technology, Qingdao, 266042 China; 2 College of Chemical Engineering, Qingdao University of Science and Technology, Qingdao, 266042 China

**Keywords:** Selective oxidation, Amino acid, Heteropolyoxometalate, 1-Propanol, Propionic acid

## Abstract

**Background:**

Propionic acid as a very valuable chemical is in high demand, and it is industrially produced via the oxo-synthesis of ethylene or ethyl alcohol and via the oxidation of propionaldehyde with oxygen. It is urgent to discover a new preparation method for propionic acid via a green route. Recyclable amino-acid-based organic–inorganic heteropolyoxometalates were first used to high-efficiently catalyse the selective oxidation of 1-propanol to propionic acid with H_2_O_2_ as an oxidant.

**Result:**

A series of amino-acid-based heteropoly catalysts using different types of amino acids and heteropoly acids were synthesized, and the experimental results showed proline-based heteropolyphosphatotungstate (ProH)_3_[PW_12_O_40_] exhibited excellent catalytic activity for the selective catalytic oxidation of 1-propanol to propionic acid owing to its high capacity as an oxygen transfer agent and suitable acidity. Under optimized reaction conditions, the conversion of 1-propanol and the selectivity of propionic acid reached 88% and 75%, respectively. Over four cycles, the conversion remained at >80%, and the selectivity was >60%. (ProH)_3_[PW_12_O_40_] was also used to catalyse the oxidations of 1-butanol, 1-pentanol, 1-hexanol, and benzyl alcohol. All the reactions had high conversions, with the corresponding acids being the primary oxidation product.

**Conclusions:**

Proline-based heteropolyoxometalate (ProH)_3_[PW_12_O_40_] has been successfully used to catalyse the selective oxidation of primary alcohols to the corresponding carboxylic acids with H_2_O_2_ as the oxidant. The new developed catalytic oxidation system is mild, high-efficient, and reliable. This study provides a potential green route for the preparation propionic acid.

## Introduction

Propionic acid, a very valuable chemical, is widely used as a preservative in the feed, food, and pharmaceutical industries and incorporated in the perfume, herbicide, and polymer industries [1, 2]. Propionic acid is industrially produced via the oxo-synthesis of ethylene or ethyl alcohol and via the oxidation of propionaldehyde with oxygen [3, 4]. However, these oxidation reactions require the use of an oil-soluble salt or a metal complex as a catalyst under harsh reaction conditions. Therefore, the development of a mild and effective synthetic method for propionic acid is of great significance.

The oxidation of primary alcohols to the corresponding carboxylic acids is one of the most important transformations in organic chemistry [5–9]. Traditionally, 1-propanol can be oxidised to propionic acid by using inorganic oxidants, such as chromate and potassium permanganate, which are expensive and generate a large amount of hazardous waste. An alternative route to the oxidation of 1-propanol using environment-friendly and cheap oxidants is preferable. Hydrogen peroxide (H_2_O_2_) has received considerable attention as a green oxidant over the past several decades owing to its easy availability, mild oxidation conditions, and single by-product (water) [10–13].

Due to their high capacity as oxygen transfer agents [14, 15], polyoxometalates are characterised as efficient catalysts in oxidation reactions with O_2_ or H_2_O_2_ [16–19]. There have been some reports on the oxidation of primary alcohols using heteropolyoxometalates as catalysts [20–23]. Nonetheless, these catalysts only promote the oxidation of primary alcohols to the corresponding aldehydes. Furthermore, most related studies have involved the oxidation of benzyl alcohol as the model substrate and benzaldehyde as the primary product [24, 25]. The selective oxidation of 1-propanol to the corresponding propionic acid via a green route has not been reported in the literature.

In this paper, we present a highly selective oxidation of 1-propanol to propionic acid with high conversion, using a recyclable organic–inorganic heteropolyoxometalate as the catalyst and H_2_O_2_ as the oxidant. Inexpensive and readily available amino acid is selected as the cation [26–28]. Moreover, its weak acidity can provide a suitable catalytic environment. Amino-acid-based heteropolyoxometalates exhibit good amphiphilicity, which enhances reactivity and realises the separation and recycling of the catalyst. Among the prepared catalysts, proline-based heteropolyphosphatotungstate (ProH)_3_[PW_12_O_40_] exhibits the best catalytic activity with good recycling efficiency. The study provides a potential green route for the preparation propionic acid.

## Materials and methods

### Chemicals

L-Proline (Pro), L-aspartic acid (Asp), L-glutamic acid (Glu), and phosphotungstic acid were purchased from Shanghai McLean Biochemical Technology Co., Ltd. Phosphomolybdic acid, silicotungstic acid, ethyl acetate, anhydrous magnesium sulfate, and 30 wt% H_2_O_2_ were purchased from Sinopharm Chemical Reagent Co., LTD. 1-Propanol and other chemicals were acquired from Shanghai Biological Technology Co., Ltd.

### Preparation of catalysts

The synthesis of a proline-based catalyst, (ProH)_3_[PW_12_O_40_], was chosen as an example. A total of 0.015 mol L-proline and 10 mL of deionised water were added to a 50-mL one-neck flask. The temperature was increased to 60 °C in a water bath; 0.05 mol of phosphotungstic acid was slowly dropped into 10 mL of an aqueous solution while stirring. The mixture was reacted at 60 °C for 24 h. After the reaction, water was removed by rotary evaporation, and the residue was further dried in a blast drying oven to obtain a white solid catalyst (ProH)_3_[PW_12_O_40_]. The synthetic method for other catalysts was similar to that of (ProH)_3_[PW_12_O_40_].

### Catalytic tests

The reaction was carried out in a 25-mL three-neck flask fitted with a reflux condenser tube. Then, 10 mmol of 1-propanol and an appropriate amount of catalyst were added to the flask. The mixture was stirred for 10 min in a 60 °C water bath, and 30 mmol of 30 wt% H_2_O_2_ solution was slowly added; the reaction was continued for 6 h at 60 °C. After the reaction, the catalyst was separated by centrifugation and reused after drying. The reaction solution was extracted three times with ethyl acetate, and the upper organic phase was combined for qualitative and quantitative analysis by a gas chromatograph with an FID detector. The lower water phase was titrated with 0.05 M NaOH solution for an integrated quantitative analysis of propionic acid.

## Results and discussion

### Catalyst Characterization

**Fig. 1:**
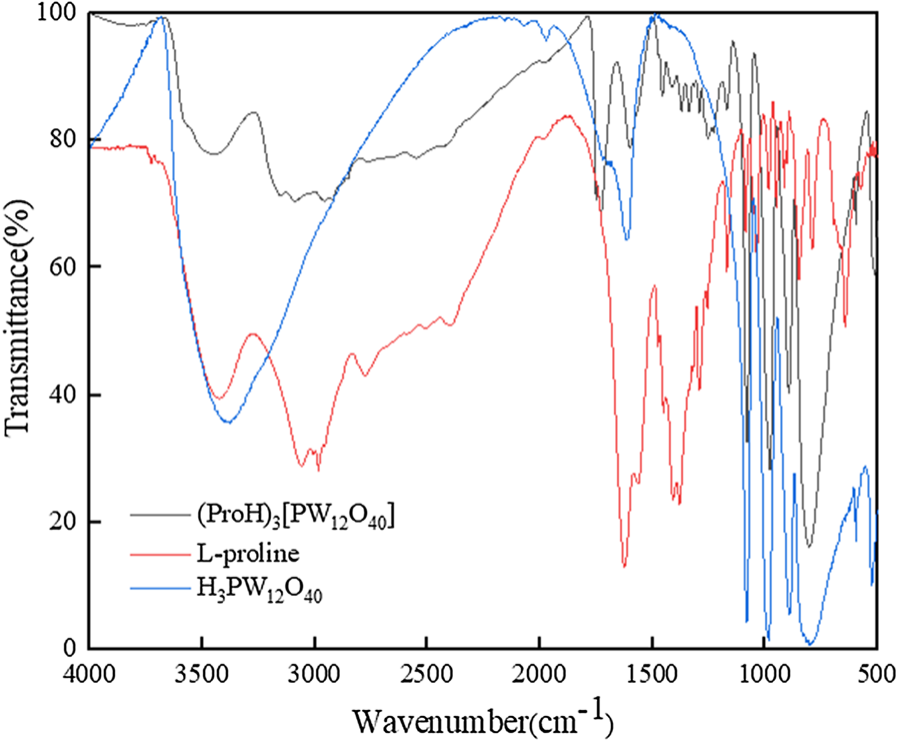
FT-IR spectra of L-proline, H_3_PW_12_O_40_ and (ProH)_3_[PW_12_O_40_]

The infrared spectra of L-proline, H_3_PW_12_O_40_, and (ProH)_3_[PW_12_O_40_] are shown in Fig. 1. The infrared spectrum of H_3_PW_12_O_40_ shows characteristic peaks at 1082 cm^−1^, 988 cm^−1^, 896 cm^−1^, and 805 cm^−1^, attributable to the stretching vibrations of P-O_a_, W = O_d_, W-O_b_-W, and W-O_c_-W, respectively, characteristics of the typical Keggin structure of heteropoly acid [29]. The primary absorption bands of (ProH)_3_[PW_12_O_40_] suggest that the Keggin structure of the heteropoly anion was retained. In the infrared spectrum of L-proline, characteristic absorption peaks at 3400 cm^−1^, 3050 cm^−1^, and 1628 cm^−1^ were assigned to the stretching vibrations of -OH, -NH_2_^+^, and C = O, respectively [30, 31]. (ProH)_3_[PW_12_O_40_] shows a similar pattern of IR peaks; however, compared with L-proline, the (ProH)_3_[PW_12_O_40_] absorption peaks move slightly towards high wave numbers, indicating the successful synthesis of L-proline-based heteropolyoxometalate (ProH)_3_[PW_12_O_40_].

**Fig. 2:**
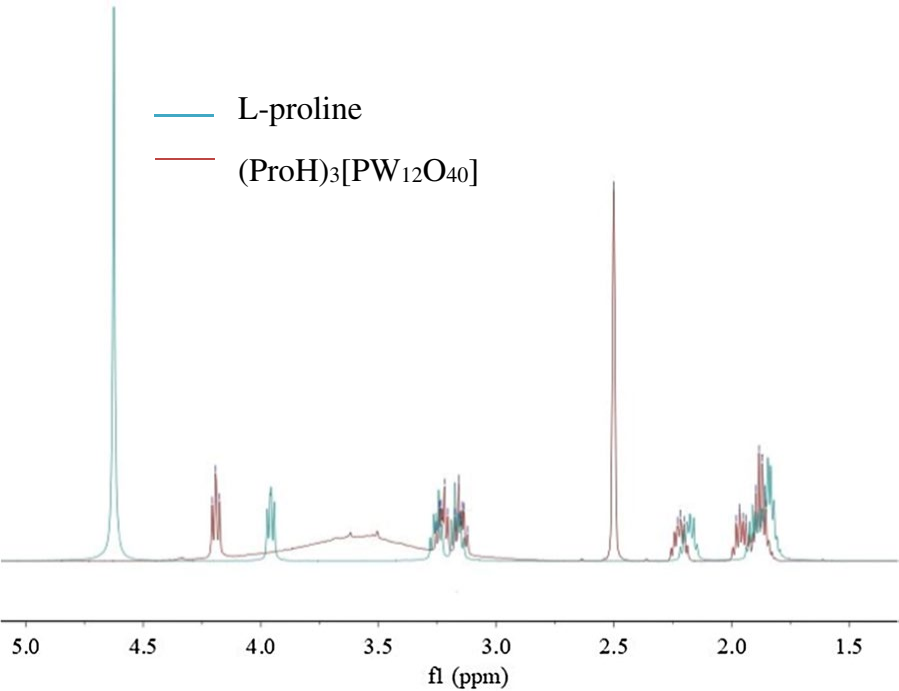
^1^H NMR spectra of L-proline and (ProH)_3_[PW_12_O_40_]

Figure 2 shows the ^1^H NMR spectra of L-proline and (ProH)_3_[PW_12_O_40_] using deuterated water and deuterated DMSO as the solvents, respectively. In the ^1^H NMR spectrum of L-proline the typical absorption peaks of hydrogen on the ring skeleton was: δ = 1.85–1.93 (m, 2H, CH_2_), 1.95–2.22 (m, unequal 2H, CH_2_), 3.15–3.25 (m, 2H, CH_2_), and 3.95 (t, J = 7.1 Hz, 1H, CH). In contrast to L-proline, the absorption peaks of (ProH)_3_[PW_12_O_40_] had the same shape, but moved toward the lower field, indicating that L-proline was successfully protonated by phosphotungstic acid. The broad peak between 3–4 ppm indicates the presence of crystalline water.

**Fig. 3:**
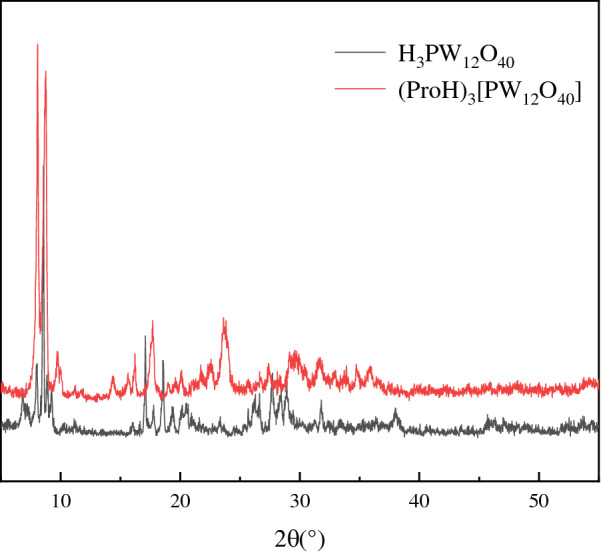
XRD patterns of H_3_PW_12_O_40_ and (ProH)_3_[PW_12_O_40_]

The XRD pattern of (ProH)_3_[PW_12_O_40_] (Fig. 3) shows obvious diffraction peaks at 2θ of 7.8°, 9.7°, 18.3°, 29.1°, 32.5°, and 37.8°, which can reflect the characteristic absorption peaks of Keggin structure [22]. However, the diffraction peaks are not exactly the same as those of H_3_PW_12_O_40_, because the hydrogen proton of H_3_PW_12_O_40_ is replaced by the proline cation.

**Fig. 4:**
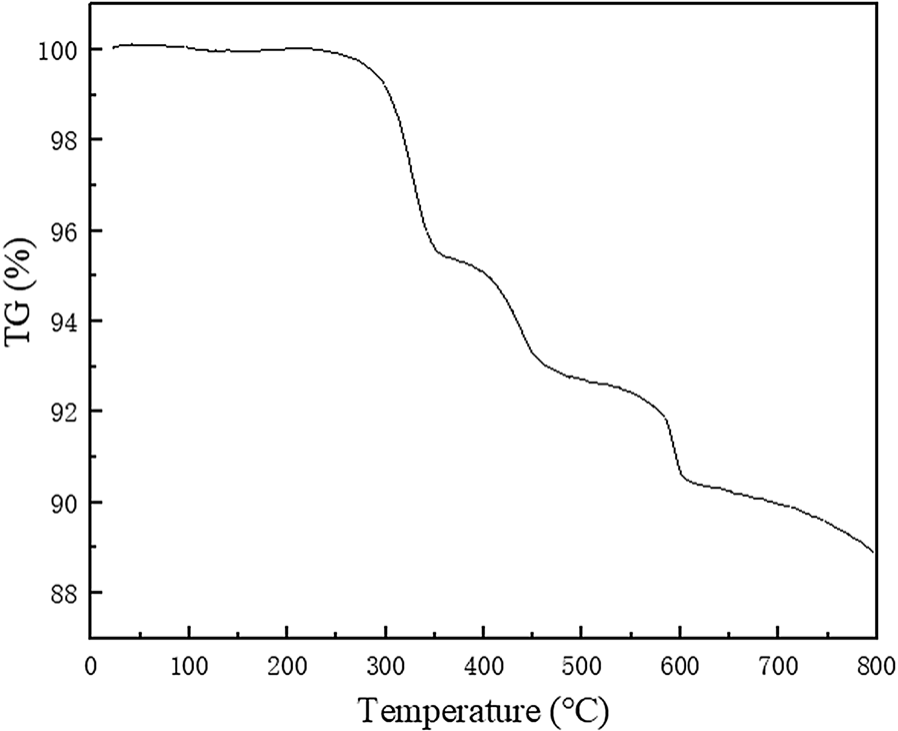
Thermogravimetric curve of (ProH)_3_[PW_12_O_40_]

The thermostability of the (ProH)_3_[PW_12_O_40_] catalyst was studied using a thermogravimetric (TG) test. The TG curve of (ProH)_3_[PW_12_O_40_] exhibits the stepwise decomposition of proline-based cations and heteropoly anions (Fig. 4). The first decomposition peak appears above 270 °C, suggesting that the catalyst has very high thermostability.

### Catalytic activity of different catalysts

From entry 1 in Table 1, only a small amount of 1-propanol is oxidized by H_2_O_2_ in the absence of catalyst, and the main oxidation product is propionaldehyde. Herein we synthesised a series of amino-acid-based heteropoly catalysts using different types of amino acids and heteropoly acids to identify the best selective catalysts for the oxidation of 1-propanol to propionic acid. The activities of these catalysts for the selective oxidation of 1-propanol were fully investigated; the results are listed in Table 1. The acid strength of the catalysts was determined by 1-butylamine titration [32], and the oxidisability of the catalyst was assessed by a redox potential assay. From entries 2–4, the conversion of 1-propanol and the selectivity for propionic acid increased with increasing oxidisability of the catalyst. The catalyst with lower oxidisability primarily catalyses the H_2_O_2_ oxidation of 1-propanol to propionaldehyde. The acidity of the catalyst also greatly affects the catalytic activity (Table 1), and suitable acidity is required for obtaining propionic acid. Excessive acidity of the catalyst may promote esterification to obtain propyl propionate (Entries 4–6 and 9). Among the different amino-acid-based catalysts tested (entries 4, 7, and 8), the proline-based catalyst (ProH)_3_[PW_12_O_40_] exhibited the best catalytic activity. In summary, the catalyst with higher oxidation properties and suitable acidity is more suitable for use in the oxidation of 1-propanol to propionic acid.

**Table 1:** Catalytic performance of different catalysts^a^

Entry	Catalyst	Acid Strength /mV ^b^	Redox potential /V ^c^	Conversion /%	Yield/%	Selectivity/%			
						Propionic acid	Propion-aldehyde	Propyl propionate	Others
1	***–***	***–***	***–***	9.36	3.87	41.35	52.56	***–***	6.09
2	(ProH)_4_[SiW_12_O_40_]	446	0.06	41.71	13.68	32.80	47.85	10.91	8.44
3	(ProH)_3_[PMo_12_O_40_]	427	0.11	30.82	18.31	59.41	29.88	6.72	3.99
4	(ProH)_3_[PW_12_O_40_]	474	0.24	73.98	45.50	61.50	32.51	4.08	1.91
5	(ProH)_2_[HPW_12_O_40_]	595	0.18	65.67	41.94	63.91	25.13	7.74	3.28
6	(ProH)[H_2_PW_12_O_40_]	653	0.13	57.94	27.19	46.93	31.48	17.24	4.35
7	(AspH)_3_[PW_12_O_40_]	490	0.19	69.99	40.53	57.91	25.29	6.97	9.83
8	(GluH)_3_[PW_12_O_40_]	574	0.15	63.54	35.71	56.20	34.56	7.69	1.54
9	H_3_PW_12_O_40_	732	0.13	64.53	36.05	55.87	28.17	13.23	2.73

^a^reaction conditions: n(1-propanol) = 10 mmol, n(catalyst) = 0.1 mmol, n(30%H_2_O_2_) = 30 mmol, T = 60 °C, t = 6 h, solvent free; ^b^0.2 mmol, 25 mL acetonitrile; ^c^oxidation potential Vs Ag/AgCl

### Optimization of catalytic conditions

For the selective oxidation of 1-propanol catalysed by (ProH)_3_[PW_12_O_40_], various reaction conditions were screened to obtain the optimised conditions that gave propionic acid in greater yields.

**Fig. 5:**
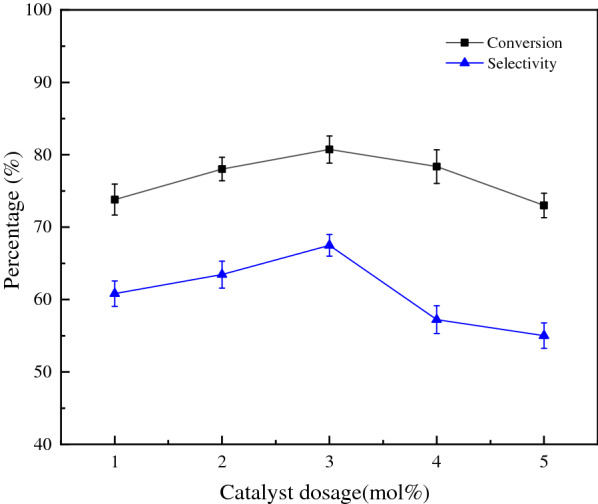
Influence of catalyst dosage on reaction (n(1-propanol) = 10 mmol, n(H_2_O_2_) = 30 mmol, T = 60 °C, t = 6 h)

Increased catalyst dosage is shown to increase the conversion and selectivity of propionic acid; this effect is limited to catalyst dosages of up to 3 mol% (Fig. 5). When the catalyst dosage was further increased, both the conversion and selectivity of propionic acid decreased, possibly because too much catalyst also improves the decomposition of hydrogen peroxide. Therefore, the best catalyst dosage was 3 mol%.

**Fig. 6:**
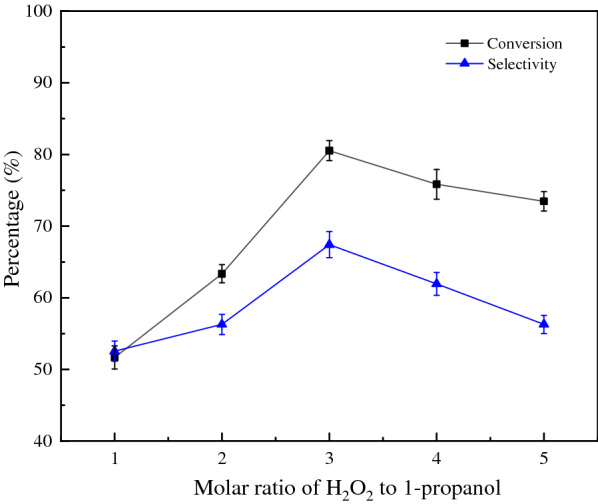
Influence of oxidant dosage on reaction (n(1-propanol) = 10 mmol, n(catalyst) = 0.3 mmol, T = 60 °C, t = 6 h)

The oxidant dosage has a significant effect on the reaction. Figure 6 shows that propionic acid yield increases with increasing amount of hydrogen peroxide and reaches a maximum when the molar ratio of H_2_O_2_ to 1-propanol is 3:1. Additional aqueous H_2_O_2_ dilutes the concentration of the substrate and the catalyst, resulting in low conversion and selectivity of propionic acid.

**Fig. 7:**
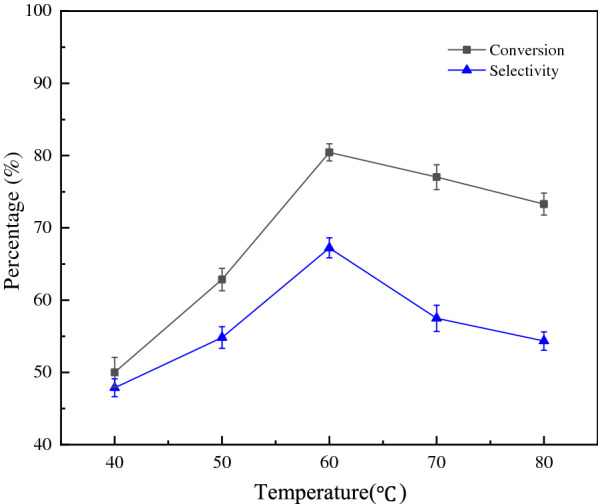
Influence of temperature on reaction (n(1-propanol) = 10 mmol, n(H_2_O_2_) = 30 mmol, n(catalyst) = 0.3 mmol, t = 6 h)

The influence of temperature on the reaction is shown in Fig. 7. The conversion and selectivity of propionic acid increased as the reaction temperature was increased from 40 from 60 °C. Nevertheless, a decrease trend for conversion and selectivity is found after 60 °C due to the decomposition of H_2_O_2_ at higher temperatures, resulting in low yields of propionic acid.

**Fig. 8:**
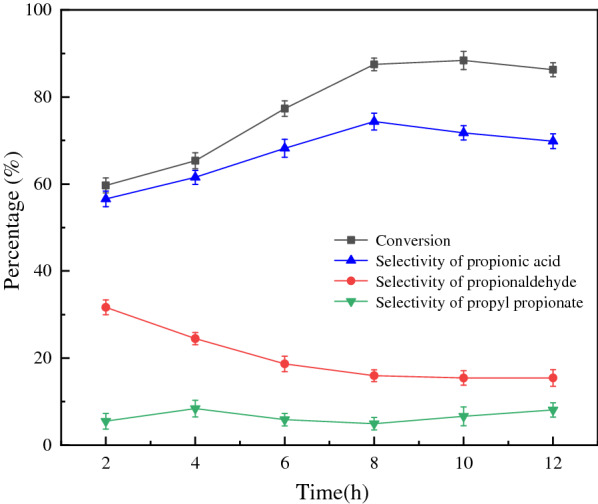
Influence of time on reaction (n(1-propanol) = 10 mmol, n(H_2_O_2_) = 30 mmol, n(catalyst) = 0.3 mmol, T = 60 °C)

Figure 8 shows the kinetic curves of the reaction at different reaction times. The conversion and selectivity of propionic acid increased significantly upon prolonged the reaction time from 2 to 8 h. After 8 h, the conversion of 1-propanol remained basically unchanged, and the selectivity of propionic acid began to decrease slightly, because the long reaction time facilitates the esterification of the formed propionic acid with 1-propanol and increases the by-product propyl propionate.

**Fig. 9:**
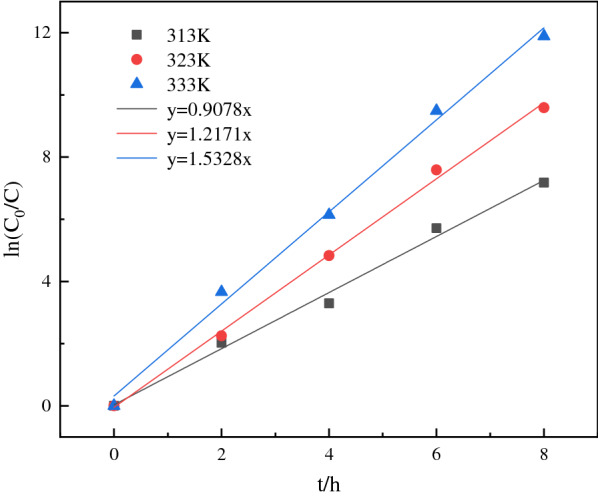
Reaction rate constants at different temperatures

Since the amount of oxidant is excessive, the reaction rate is supposed to only be decided by the concentration of 1-propanol. Reaction rate constants at different temperatures were shown in Fig. 9. According to Arrhenius equation [33], the calculated apparent activation energy is 22.64 kJ/mol.

In summary, the optimum reaction conditions for the preparation of propionic acid were as follows: catalyst amount of 3 mol%, n(H_2_O_2_):n(1-propanol) ratio of 3:1, reaction temperature of 60 °C, and reaction time of 8 h. Under the selected optimised conditions, the conversion of 1-propanol was 88%, and the selectivity of propionic acid reached 75%.

### Proposed catalytic mechanism

**Scheme 1:**
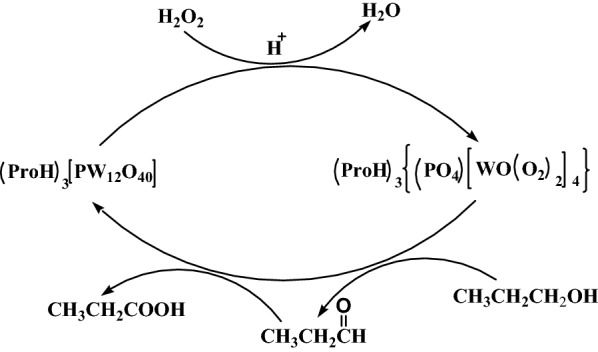
Proposed mechanism of the catalytic oxidation of 1-propanol

According to the reaction results obtained herein and those reported previously [34], the proposed catalytic mechanism for the oxidation of 1-propanol catalysed by (ProH)_3_[PW_12_O_40_] is shown in Scheme 1. In the oxidation process, the catalyst (ProH)_3_[PW_12_O_40_] first reacts with H_2_O_2_, and the heteropoly anion of the catalyst is depolymerized to a smaller active peroxygen intermediate. This intermediate (the active centre) subsequently oxidises the substrate 1-propanol to propionaldehyde, which is further oxidised to propionic acid. The kinetic curves of the reaction at different reaction times (Fig. 8) revealed that with increasing reaction time, the selectivity of propionaldehyde decreased and the selectivity of propionic acid gradually increased, indicating that 1-propanol was first oxidised to propionaldehyde and then to propionic acid. Nonetheless, the formation of propionaldehyde and its oxidation can simultaneously proceed. After the oxidation reaction, the active intermediate loses active oxygen and re-polymerises into the original Keggin structure.

### Reuse of the catalyst

**Fig. 10:**
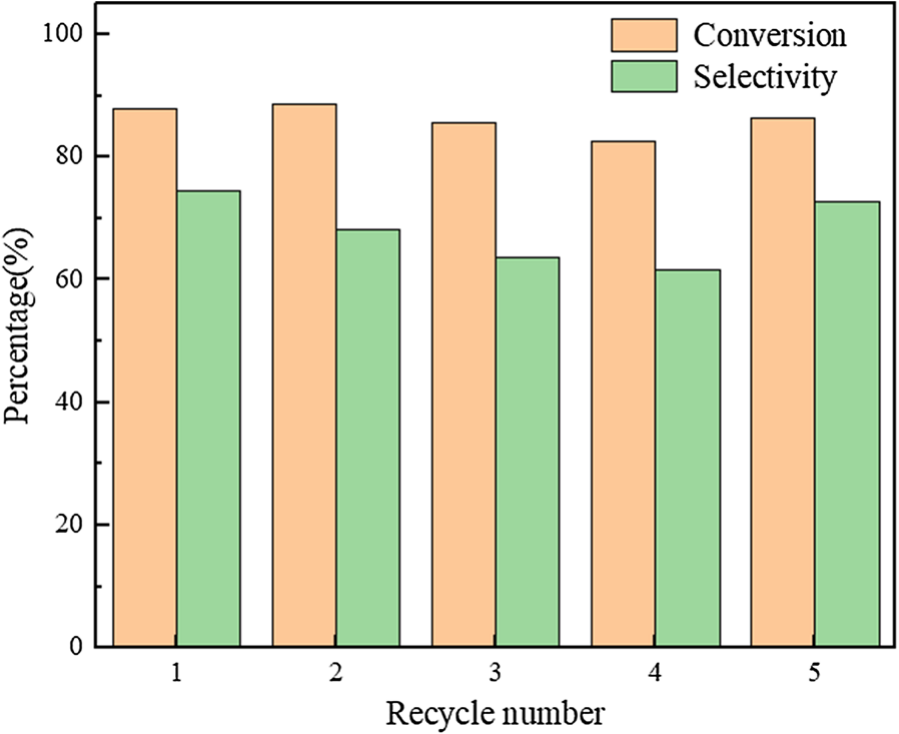
Reuse performance of the catalyst

**Fig. 11:**
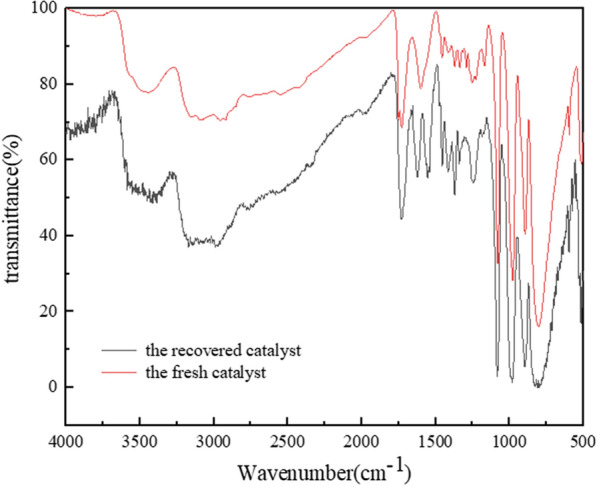
FT-IR spectra of the fresh and recovered catalysts

**Fig. 12:**
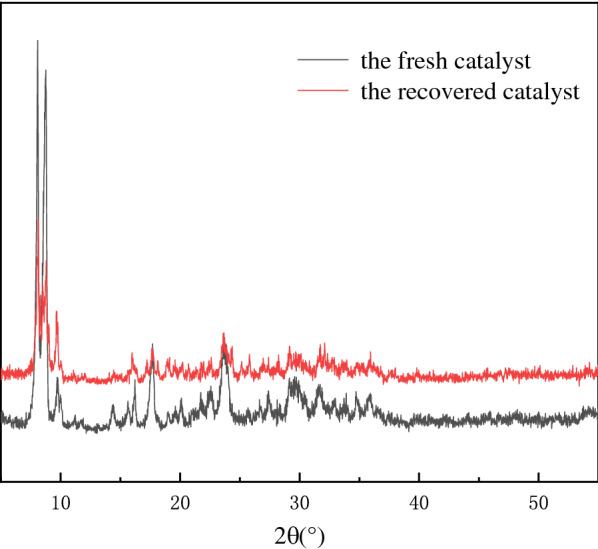
XRD patterns of the fresh and recovered catalysts

After the reaction, the catalyst was recovered by centrifugation. Figure 10 shows the cycling performance of (ProH)_3_[PW_12_O_40_] for catalysing the oxidation of 1-propanol under the optimised conditions. Over the first four cycles, the conversion of 1-propanol and the selectivity of propionic acid gradually declined. However, the conversion remained at >80%, and the selectivity was >60%. After four cycles, the recovered catalyst was characterised by FT-IR (Fig. 11) and XRD (Fig. 12). Compared with the fresh catalyst, the structure of the recovered catalyst was not destroyed in the first four cycles, indicating good stability. The decrease in catalytic activity may be due to a slight loss of the catalyst during separation. For the fifth cycle, an equivalent amount of the lost catalyst was added, and catalytic activity was restored (Fig. 10).

### Substrate expansion

The catalytic performance of the (ProH)_3_[PW_12_O_40_] catalyst in the oxidation of other primary alcohols was also investigated; the results are shown in Table 2. The (ProH)_3_[PW_12_O_40_] catalyst exhibits good catalytic activity for various aliphatic primary alcohols and aromatic benzyl alcohol. All the reactions have high conversions, with the corresponding acids being the primary oxidation product. The selectivity of acids can be further improved by optimising the catalytic conditions. Therefore, (ProH)_3_[PW_12_O_40_] as a catalyst for the selective oxidation of primary alcohols to the corresponding acids by H_2_O_2_ has good substrate adaptability.

**Table 2:** The oxidation of different primary alcohols catalysed by (ProH)_3_[PW_12_O_40_]

Entry	Substrate	Product	Conversion /%	Yield /%	Selectivity /%
1	1-propanol	Propionic acid	87.96	65.62	74.61
2	1-butanol	Butyric acid	85.56	61.38	71.74
3	1-pentanol	Pentanoic acid	82.61	54.03	65.76
4	1-hexanol	Hexanoic acid	80.23	50.87	63.41
5	Benzyl alcohol	Benzyl acid	81.02	63.09	77.87

Reaction conditions: n(substrate) = 10 mmol, n(catalyst) = 0.3 mmol, n(30%H_2_O_2_) = 30 mmol, 60 °C, 8 h

## Conclusions

Proline-based heteropolyoxometalate (ProH)_3_[PW_12_O_40_] has been successfully used to catalyse the selective oxidation of 1-propanol toward propionic acid with H_2_O_2_ as the oxidant. The conversion of 1-propanol and the selectivity of propionic acid reached 88% and 75%, respectively. The excellent catalytic activity of (ProH)_3_[PW_12_O_40_] is attributed to its high capacity as an oxygen transfer agent with a suitable acidity. (ProH)_3_[PW_12_O_40_] also exhibited good recycling efficiency. This study provides a new preparation method for propionic acid via a green route, and the developed catalyst shows immense potential for the selective oxidation of other primary alcohols to the corresponding carboxylic acids with H_2_O_2_ as an oxidant.

## Data Availability

All data generated or analysed during this study are included in this published article.
